# scDIOR: single cell RNA-seq data IO software

**DOI:** 10.1186/s12859-021-04528-3

**Published:** 2022-01-06

**Authors:** Huijian Feng, Lihui Lin, Jiekai Chen

**Affiliations:** 1 Center for Cell Lineage and Atlas, Bioland Laboratory (Guangzhou Regenerative Medicine and Health Guangdong Laboratory), Guangzhou, 510005 People’s Republic of China; 2 CAS Key Laboratory of Regenerative Biology, Guangdong Provincial Key Laboratory of Stem Cell and Regenerative Medicine, Guangzhou Institutes of Biomedicine and Health, Chinese Academy of Sciences, Guangzhou, 510530 People’s Republic of China; 3 Joint School of Life Sciences, Guangzhou Institutes of Biomedicine and Health, Chinese Academy of Sciences, Guangzhou Medical University, Guangzhou, 511436 People’s Republic of China; 4 Centre for Regenerative Medicine and Health, Hong Kong Institute of Science and Innovation, Chinese Academy of Sciences, Hong Kong SAR, People’s Republic of China

**Keywords:** Single-cell, Data IO, HDF5

## Abstract

**Background:**

Single-cell RNA sequencing is becoming a powerful tool to identify cell states, reconstruct developmental trajectories, and deconvolute spatial expression. The rapid development of computational methods promotes the insight of heterogeneous single-cell data. An increasing number of tools have been provided for biological analysts, of which two programming languages- R and Python are widely used among researchers. R and Python are complementary, as many methods are implemented specifically in R or Python. However, the different platforms immediately caused the data sharing and transformation problem, especially for Scanpy, Seurat, and SingleCellExperiemnt. Currently, there is no efficient and user-friendly software to perform data transformation of single-cell omics between platforms, which makes users spend unbearable time on data Input and Output (IO), significantly reducing the efficiency of data analysis.

**Results:**

We developed scDIOR for single-cell data transformation between platforms of R and Python based on Hierarchical Data Format Version 5 (HDF5). We have created a data IO ecosystem between three R packages (Seurat, SingleCellExperiment, Monocle) and a Python package (Scanpy). Importantly, scDIOR accommodates a variety of data types across programming languages and platforms in an ultrafast way, including single-cell RNA-seq and spatial resolved transcriptomics data, using only a few codes in IDE or command line interface. For large scale datasets, users can partially load the needed information, e.g., cell annotation without the gene expression matrices. scDIOR connects the analytical tasks of different platforms, which makes it easy to compare the performance of algorithms between them.

**Conclusions:**

scDIOR contains two modules, dior in R and diopy in Python. scDIOR is a versatile and user-friendly tool that implements single-cell data transformation between R and Python rapidly and stably. The software is freely accessible at https://github.com/JiekaiLab/scDIOR.

## Background

The scale of single-cell RNA sequencing (scRNA-seq) data has grown from tens of single cells up to millions of single cells per study during the past 10 years [1]. Characterizing the heterogeneity is a rate-limiting step in identifying rare cell types. Scaling up the sequencing cell number from complex samples facilitates the coverage of various proportions of cell types [2–5]. However, the size of data has also increased rapidly with the increase of cell numbers, resulting in time-consuming data input–output (IO). In addition, although the integrated utilization of the different platforms’ tools is needed for many single-cell analytical tasks, the data structures of these platforms are considerably different, making it difficult to transform data between them. Current analytics tools for scRNA-seq data are written in a variety of programming languages, most popularly R and Python [1]. Prevailing single-cell platforms such as Seurat [6, 7], Scran [8], Monocle [3, 9], and Scanpy [10] provide numerous analytics tools. Different platforms have their specialty. Seurat v3 is the recommended method for batch integration [11]; Scran is widely known for normalization of the non-full-length dataset [12]; Monocle is expert at the reconstruction of the cell fate trajectory [3, 9]; Scanpy is scalable in dealing with large datasets of more than one million cells and flexible with strong functional expansion [10]. Therefore, the use of different platforms is necessary to fully utilize the advantages of different algorithms and techniques to mine biological phenomena. However, the lack of a method that allows scRNA-seq data transformation across different platforms makes data analysis difficult for biologists.

Recent advances in spatially resolved transcriptomics have greatly expanded the knowledge of complex multicellular organisms [13]. Several advanced techniques have been developed to integrate gene expression data with spatial information. Since Seurat and Scanpy can be used to deal with both the scRNA-seq and spatial transcriptomics data. A convenient tool for transformation of multiple omics data between platforms is needed.

Although the programming language and class of the tools are different, there is similar hierarchical information for scRNA-seq data and spatial data in these platforms, containing the matrix of expression data, cell annotation, gene annotation, dimension reduction in common. Here we used Hierarchical Data Format (HDF5), a high-performance data management and storage suite (https://www.hdfgroup.org/solutions/hdf5) to store this information. HDF5 is a software that runs on a range of computational platforms with great access time and storage space optimizations. We find that the IO efficiency of HDF5 format is much higher than the tab-separated format. Specially, we used the hdf5r package for R-based platforms and the h5py package for Python-based platforms.

## Implementation

**Fig. 1:**
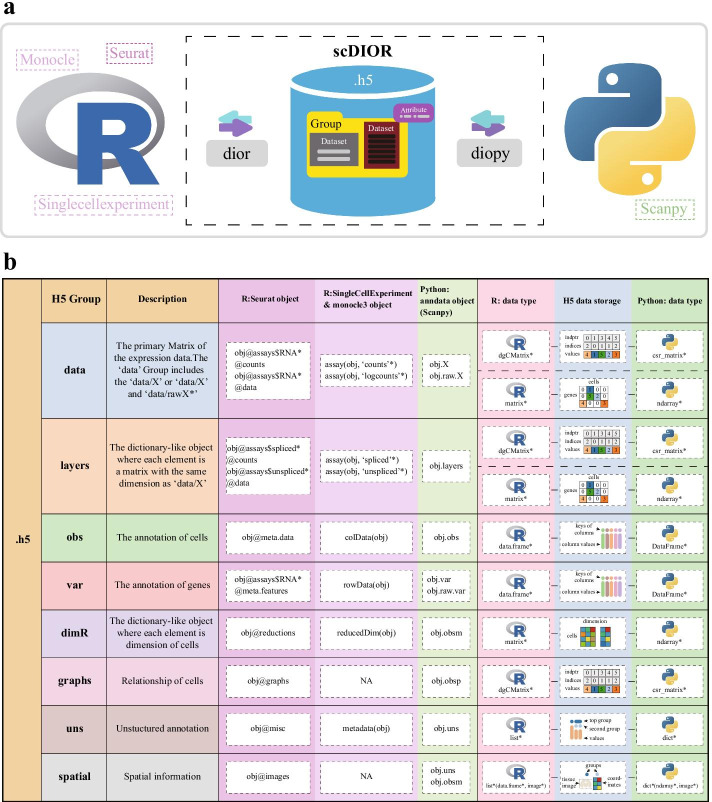
scDIOR workflow. **a** scDIOR contains two modules, where dior and diopy. scDIOR implements the single-cell data IO between R (Seurat, SingleCellExperiment and Monocle) and Python (Scanpy) through the hierarchical construction of HDF5 group, HDF5 dataset, and HDF5 attribute; **b** scDIOR create the ‘.h5’ file containing the groups of data, layers, obs, var, dimR, graphs, uns, and spatial. Column ‘Description’ is brief description of the group information. Column ‘R:Seurat object’, ‘R: SingleCellExperiment & Monocle3 object’, and ‘Python: anndata object (Scanpy)’ represents the groups corresponding to the slot of data in the Seurat, Singlecellexperiment(Monocle3), and anndata object. Column ‘R: data type’, ‘H5 data type’, and ‘Python: data type’ represents the common storage format of R and Python in ‘.h5’ file. The dgCMatrix means R Matrix package ‘dgCMatrix’ object. The matrix means R base package ‘matrix’ object. The data.frame means R base package ‘data.frame’ object. The list means R base package ‘list’ object. The csr_matrix means Python SciPy module ‘scipy.sparase.csr.csr_matrix’ object. The ndarray means Python NumPy module ‘numpy.ndarray’ object. The DataFrame means Python Pandas module ‘pandas.core.frame.DataFrame’ object. The dict means Python ‘dict’ object

scDIOR contains two modules, dior for R and diopy for Python. The data transformation was implemented by a ‘.h5’ file of HDF5 format, which harmonizes the different data types between R and Python. The different aspects of single-cell information were stored in HDF5 group with dataset (Fig. 1a). scDIOR creates 8 HDF5 groups to store core single-cell information, including data, layers, obs, var, dimR, graphs, uns and spatial. Each group contains one or more datasets of group content, e.g., the value of gene expression matrix, the column values of cell annotation data frame, or the unstructured annotation for plotting. The attributes record the datatype of the group or datasets. The attributes are important to indicate the method to store and restore the data. For example, the matrix with attribute ‘Array’ is stored directly using HDF5 method, while the matrix with attribute ‘SparseMatrix’ is stored by three vectors of indices, indptr and values with three datasets.

The HDF5 group ‘data’ includes ‘data/X’ and ‘data/rawX’ (option), which are the secondary groups under the group ‘data’ (Fig. 1b). The group ‘data’ stores the primary matrix of gene expression of Seurat, SingleCellExperiment, and anndata objects (Scanpy). scDIOR implemented the unification of the sparse matrices between Compressed Sparse Column (CSC) format (R: Matrix dgCMatrix object) and Compressed Sparse Row (CSR) format (Python: SciPy scipy.sparse.csr.csr_matrix). Group ‘data’ converts them into the three one-dimensional arrays with three HDF5 datasets (indptr, indices, values) (Fig. 1b). For $m\times p$ CSC(CSR) matrix $M$ with $n$ nonzero of entries, the array ‘values’ is an array of one dimension that corresponds to the collapsing of $n$ entries by columns(row) in $M$. The array ‘indices’ is an array of the same length with ‘values’, which represents the row(column) indices of each entry in array ‘values’. The array ‘indptr’ is an $p+1$($m+1$) array, each the two adjacent entries define the range of values in array ‘values’ and indices belonging to a particular column(row). scDIOR can also transform the dense matrices between base matrix object of R and numpy.ndarray object of Python by utilizing the group ‘data’ (Fig. 1b).

The HDF5 group ‘layers’ is used to save the matrices with the same dimension as group ‘data/X’ (Fig. 1b), e.g., the spliced and unspliced UMI counts used for RNA velocity pipeline.

Since the cell and gene annotations are recorded in data frames, scDIOR creates the HDF5 group ‘obs’ and ‘var’ to save them (Fig. 1b). The columns of data frame are split into multiple arrays and stored in multiple HDF5 datasets. The key name of dataset corresponds to the column name, and the value of the dataset corresponds to the column value. scDIOR also creates a group ‘obs/category’ or ‘var/category’ to save the categories or levels of column values. Due to this design, the categories or factor can be preserved when transforming the data from Python to R, and vice versa.

The group ‘dimR’ stores the reduced dimensional information of cells, e.g., PCA and UMAP. The group ‘graphs’ stores the affinity matrices of cells in sparse matrix format, e.g., k-nearest neighbors matrix. The group ‘uns’ stores the unstructured annotation of the data, e.g., the colors of batches. The group ‘spatial’ stores the spatial transcriptome information, e.g., the image and location of the sampling spots.

## Results

We developed scDIOR for single-cell data IO between R and Python, which contained two modules, dior and diopy. scDIOR implements the unification of data format of different single-cell analytics platforms.

**Fig. 2:**
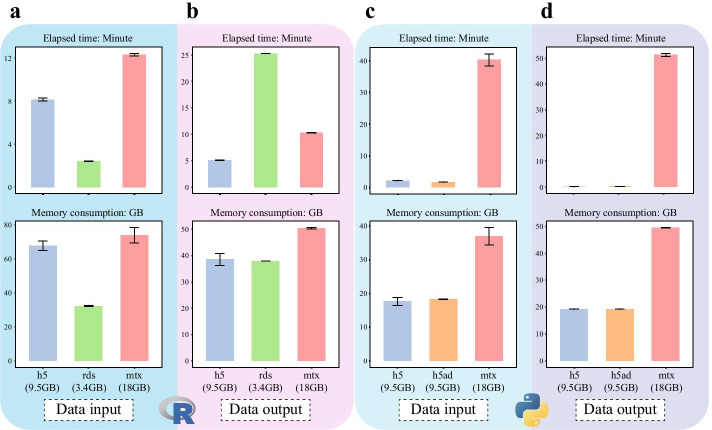
The IO performance of scDIOR. **a**, **b** The IO performance was evaluated between h5, rds and mtx in R. **a** For the elapse time of data reading and **b** for the elapse time of data storing; **c**, **d** The IO performance was evaluated between h5, h5ad and mtx in Python. **c** For the elapse time of data reading and **d** for the elapse time of data storing

We evaluated the performance of available data transformation software between R and Python. We used the Cao2019 dataset [3] with 1,943,759 cells and 26,157 genes as the standard input. The data was transformed into three data format, ‘.mtx’ (Matrix Market format of COOrdinate sparse matrix), ‘.rds’ (R object for Seurat), ‘.h5ad’ (HDF5 file for Scanpy) and ‘.h5’ (HDF5 file for scDIOR). The corresponding file sizes are 18 GB, 3.4 GB, 9.5 GB, 9.5 GB. The ‘.rds’ and ‘.h5ad’ were designed for and can be used by R or Python, respectively, while ‘.h5’ can be used for both languages. In R environment, we recorded the IO speed and peak memory cost of ‘.h5’, ‘.rds’ and ‘.mtx’. The elapsed time of reading ‘.h5’ is about 1.5 times shorter than ‘.mtx’ (Fig. 2a, top panel). The elapsed time of reading ‘.rds’ is much shorter than ‘.h5’ and ‘.mtx’ since the file size of ‘.rds’ is much smaller. However, the storing time of ‘.h5’ is much shorter than ‘.rds’ and ‘.mtx’. Note that the memory consumption of reading ‘.h5’ is similar to ‘.mtx’, suggesting that the HDF5 package for R could be optimized in the future (Fig. 2a, b, bottom panel). In Python environment, we tested the IO speed and memory cost of ‘.h5’,’.h5ad’,’.rds’. The elapsed time of reading and storing time of ‘.h5’ and ‘.h5ad’ was similar and much shorter than ‘.mtx’ (Fig. 2c, d, top panel). In addition, reading and storing ‘.h5’ and ‘.h5ad’ only consume half of the memory than ‘.mtx’ (Fig. 2c, d, bottom panel). The results showed that ‘.h5’ is a high-performance format that can be used to manage and transform data across different platforms.

**Fig. 3:**
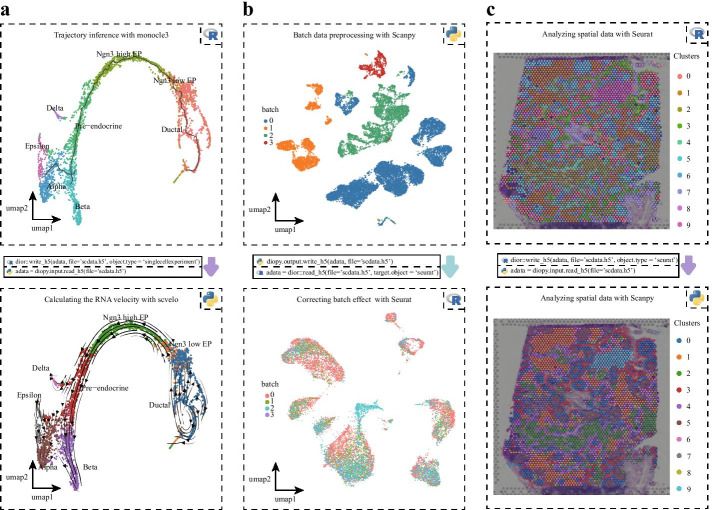
scDIOR connects the different analysis processes of different platforms. **a** The trajectory analysis results using Monocle3 in R can be easily transformed to anndata object in Python using scDIOR; **b** scDIOR links the preprocess and normalization pipeline of Scanpy and batch correction method of Seurat; **c** scDIOR can perform spatial omics data IO between Seurat and Scanpy

Different software on different platforms has specialized advantages for specific analysis tasks, making full use of their advantages will help users discover new phenomena. One can perform trajectory analysis using Monocle3 [3] in R, then transform the single-cell data to Scanpy in Python using scDIOR, such as expression profiles of spliced and unspliced, as well as low dimensional layout. The expression profile can be used to run dynamical RNA velocity analysis [14] and the results can be projected on the layout of Monocle3 (Fig. 3a). scDIOR provides an easy way to compare the trajectory analysis performance between tools. scDIOR also helps link analysis pipeline between Python and R. One can employ single-cell data preprocess and normalization method provided by Scanpy [10], and utilize batches correction method provided by Seurat [7] (Fig. 3b). In addition, scDIOR supports spatial omics data IO between R and Python platforms (Fig. 3c). These results suggest that scDIOR is a convenient and versatile software that can handle different single-cell data types and the data IO capabilities of different software on different platforms, avoiding the complicated process of intermediate data IO, and greatly improving the continuity and efficiency of analysis.

**Fig. 4:**
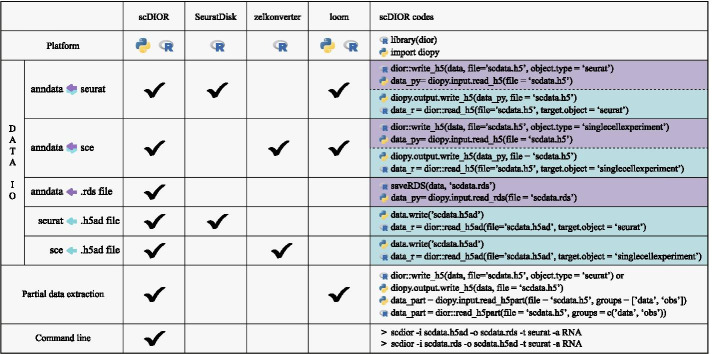
scDIOR is superior to other IO software. scDIOR implement data interconversion between anndata, Seurat, and SingleCellExperiment. scDIOR can load ‘.rds’ file in Python and ‘.h5ad’ in R. scDIOR supports partial data extraction. Data transformation can be done using command line. The reading and writing of loom files depend on the extended software

Several software for cross-platform data transformation have been proposed. We compared the characters of scDIOR, SeuratDisk, Zellkonverter and Loom (Fig. 4). The SeuratDisk(https://mojaveazure.github.io/seurat-disk/reference/SeuratDisk-package.html) is a HDF5 based R tools for interconversion between Seurat and Scanpy. Zellkonverter (http://www.bioconductor.org/packages/release/bioc/html/zellkonverter.html) is the HDF5 based Python tool that focused on the transformation between Scanpy and SingleCellExperiment. It utilizes a frozen Python environment for data storage to prevent package version incompatibility. Loom (http://loompy.org/) is the HDF5 based file format for scRNA-seq data, in which there is no interfaces between Seurat, SingleCellExperiment and Scanpy. Moreover, the use of loom files require external software, such as calling functions LoadLoom/SaveLoom implemented in SeuratDisk, calling functions import/export implemented in LoomExperiment (https://bioconductor.org/packages/release/bioc/html/LoomExperiment.html), calling functions read_loom/write_loom implemented in Scanpy. Since the method of reading and writing.loom file is designed by different labs, the cross-platform data interconversion could be difficult. SeuratDisk, ZellKonverter and Loom only support limited data objects conversion. However, the conversion between Seurat, SingleCellExperiment and Scanpy data object can be performed using scDIOR easily (Fig. 4).

scDIOR also provides the function to load ‘.rds’ file in Python, and load ‘.h5ad’ file in R directly (Fig. 4). In this scenario, the ‘.rds’ or ‘.h5ad’ will be first converted to ‘.h5’and then loaded by scDIOR. In addition, scDIOR provides easy functions for partial information extraction, by which users can load the data partially instead of the whole dataset, e.g., loading the cell annotation data frame regardless of the gene expression matrix with great size. This character of scDIOR helps save the memory and accelerate the file reading (Fig. 4). All the functions of scDIOR can be implemented in command line using only a few codes (Fig. 4).

In general, scDIOR has excellent performance in the speed of single-cell data IO, saving the time of data storage and extraction. scDIOR is convenience and versatility, which can well connect the different analysis processes of different platforms. scDIOR supports users to extract the data partially by ignoring the unused information, which saves the memory and accelerates the file reading. All the data transformation across platforms can be done by a few codes in IDE or command line. For the version control, scDIOR is widely compatible with multiple versions of SingleCellExperiment (≥ 1.8.0), Seurat (≥ v3) and Scanpy (≥ 1.4). scDIOR is an effective and user-friendly tool that will improve the utilization of advantages of different analytics platforms.

## Discussion

Single-cell datasets have become more widespread nowadays. Although scRNA-seq is the most common technique to delineate the tissue heterogeneity and cell fate determination, other methods detail processes such as in situ position, chromatin accessibility and methylation. At the same time, the number of tools designed to analyze these data has dramatically increased. However, the programming languages and designs used to develop these analytics tools are diverse. The platform difference is now becoming a challenge for researchers to transform their dataset and integrate analytical pipelines across platforms. It costs the researchers much time to tidy up the data before they employ the tools with the different data structure. scDIOR provides a lightweight but powerful tool for researchers to transform scRNA-seq dataset across platforms of R and Python. Although scDIOR covers the major scRNA-seq analytics platforms, such as Scanpy, Seurat and SingleCellExperiment, other frequently used platforms with various omics and various functions could be integrated into this framework in the future.

## Conclusion

We developed scDIOR for single-cell data IO between R and Python, which implements the unification of single-cell core dataset and cross-platform data IO (https://github.com/JiekaiLab/scDIOR). The HDF5 based format accommodates the increasing scale of single-cell data profiles. All the information, such as expression matrices, metadata, dimensional reduction, similarity graph, and spatial transcriptomes can be saved in ‘.h5’ and loaded using only a few codes. The ‘.h5’ is good data carrier that can be used to transform data to a specific data object in R and Python. We also provide methods to reciprocal conversion between data objects in the command line directly. The fast and user-friendly software will facilitate the utilization of computational methods in different platforms.

## Availability and requirements

Project name: scDIOR

Project home page: https://github.com/JiekaiLab/scDIOR

Operating systems: Platform independent

Programming language: R, Python

Other requirements: R 3.6 or higher, Python 3.6 or higher

License: GPL

Any restrictions to use by non-academics: license needed

## Data Availability

Data in Fig. 2 was downloaded in the URL: https://oncoscape.v3.sttrcancer.org/atlas.gs.washington.edu.mouse.rna/downloads Data in Fig. 3a was curated by Scanpy. The data can be loaded by the following codes: >>>import scanpy as sc >>>adata = sc.read('data/pancreas.h5ad', backup_url = 'https://www.dropbox.com/s/qj1jlm9w10wmt0u/pancreas.h5ad?dl=1') Data in Fig. 3b was curated by 10x Genomics official website, downloaded in the URL: https://support.10xgenomics.com/spatial-gene-expression/datasets Data in Fig. 3c was curated by scvelo. The data can be loaded by the following codes: >>>import scvelo as scv >>> adata = scv.datasets.pancreas()

## Abbreviations

IO: Input and Output
scRNA-seq: Single-cell RNA sequencing
HDF5: Hierarchical Data Format Version 5
CSC: Compressed Sparse Column
CSR: Compressed Sparse Row
IDE: Integrated Development Environment

## Availability of codes

scDIOR is available and open source at github (https://github.com/JiekaiLab/scDIOR).
